# 6-Methyl­ideneandrost-4-ene-3,17-dione

**DOI:** 10.1107/S1600536812013207

**Published:** 2012-03-31

**Authors:** L. C. R. Andrade, M. J. M. de Almeida, F. M. Fernandes Roleira, C. L. Varela, E. J. Tavares da Silva

**Affiliations:** a CEMDRX, Department of Physics, Faculty of Sciences and Technology, University of Coimbra, P-3004-516 Coimbra, Portugal; b Center for Pharmaceutical Studies, Pharmaceutical Chemistry Group, Faculty of Pharmacy, University of Coimbra, P-3000-548 Coimbra, Portugal

## Abstract

In the title compound, C_20_H_26_O_2_, which is the 6-methyl­ene derivative of androstenedione and a synthetic percursor of exemestane, the steroid *A* ring approximates to a sofa (or envelope) conformation, with the methyl­ene group adjacent to the link to the *B* ring lying out of the plane of the other atoms. The *B* and *C* rings have slightly flattened chair conformations and the *D* ring is an envelope, with the CH group forming the flap. In the crystal, mol­ecules are linked by two distinct C—H⋯O hydrogen bonds, involving acidic H atoms close to C=C and C=O double bonds.

## Related literature

For the synthesis of the title compound, see: Annen *et al.* (1982 ▶). For exemestane aromatase inhibitor potency, see: Furr (2006 ▶). For elucidation of structural requirements needed to achieve anti­tumor activity, see: Cepa *et al.* (2005 ▶). For puckering parameters, see: Cremer & Pople (1975 ▶) and for asymmetry parameters, see: Duax & Norton (1975 ▶); Altona *et al.* (1968 ▶). For reference bond-length data, see: Allen *et al.* (1987 ▶).
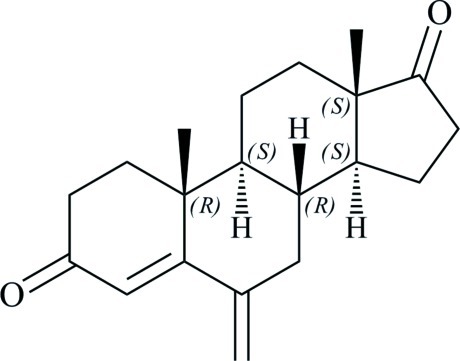


## Experimental

### 

#### Crystal data

C_20_H_26_O_2_
*M* *_r_* = 298.41
Monoclinic, 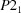

*a* = 9.2343 (4) Å
*b* = 8.7162 (4) Å
*c* = 11.0798 (5) Å
β = 108.197 (2)°
*V* = 847.19 (7) Å^3^
*Z* = 2
Mo *K*α radiation
μ = 0.07 mm^−1^
*T* = 293 K
0.24 × 0.17 × 0.05 mm

#### Data collection

Bruker APEX CCD diffractometer
Absorption correction: multi-scan (*SADABS*; Sheldrick, 2000 ▶) *T*_min_ = 0.835, *T*_max_ = 0.996
18560 measured reflections
1979 independent reflections
1433 reflections with *I* > 2σ(*I*)
*R*_int_ = 0.036

#### Refinement

*R*[*F* ^2^ > 2σ(*F* ^2^)] = 0.040
*wR*(*F* ^2^) = 0.100
*S* = 1.02
1979 reflections
201 parameters
1 restraint
H-atom parameters constrained
Δρ_max_ = 0.14 e Å^−3^
Δρ_min_ = −0.13 e Å^−3^

### 

Data collection: *SMART* (Bruker, 2003) ▶; cell refinement: *SAINT* (Bruker, 2003) ▶; data reduction: *SAINT* ▶; program(s) used to solve structure: *SHELXS97* (Sheldrick, 2008 ▶); program(s) used to refine structure: *SHELXL97* (Sheldrick, 2008 ▶); molecular graphics: *PLATON* (Spek, 2009 ▶); software used to prepare material for publication: *SHELXL97*.

## Supplementary Material

Crystal structure: contains datablock(s) global, I. DOI: 10.1107/S1600536812013207/hb6686sup1.cif

Structure factors: contains datablock(s) I. DOI: 10.1107/S1600536812013207/hb6686Isup2.hkl

Additional supplementary materials:  crystallographic information; 3D view; checkCIF report

## Figures and Tables

**Table 1:** Hydrogen-bond geometry (Å, °)

*D*—H⋯*A*	*D*—H	H⋯*A*	*D*⋯*A*	*D*—H⋯*A*
C2—H2*A*⋯O17^i^	0.97	2.43	3.345 (3)	158
C66—H66*A*⋯O3^ii^	0.93	2.47	3.365 (3)	163

Symmetry codes: (i) 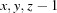; (ii) 
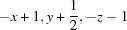
.

**Fig. 1.**
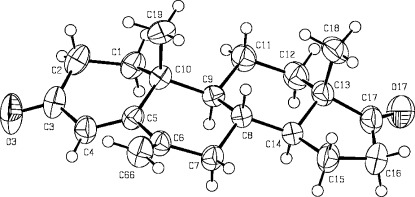
A view of the title compound. Displacement ellipsoids are drawn at the 50% level.

## supplementary crystallographic information

### Comment

The title compound is the 6-methylene derivative of androstenedione, the natural substrate of aromatase, and is a key synthetic precursor of exemestane, the most potent steroid aromatase inhibitor clinically used in the breast cancer treatment (Furr, 2006). Following our work on the determination of several androstane structures of potential aromatase inhibitors and intermediates of their syntheses, the X-ray analysis of compound (I) aims to contribute to the elucidation of structural requirements needed to achieve antitumor activity (Cepa *et al.*, 2005). From the single-crystal diffraction measurements one can conclude that bond lengths are within normal values (Allen *et al.*, 1987) with an average C*_sp_*^3^–C*_sp_*^3^ bond length of 1.534 (13) Å. Due to the C4═C5 double bond ring A addopts a 1α–sofa conformation, slightly distorted towards a 1α,2β–halfchair one [asymmetry parameters (Duax and Norton, 1975): ΔC_s_(1)=7.8 (3), ΔC_2_(1,2)=17.5 (3) and ΔC_2_(2,3)=51.4 (4)°]. Rings B and C have slightly flattened chair conformations evidenced by average torsion angle values of 50 (3) and 55 (3)°, respectively. The five member D ring assumes a 14α–envelope conformation [puckering parameters (Cremer and Pople, 1975): q_2_=0.414 (3) Å and φ_2_=211.3 (4)°; pseudo-rotation (Altona *et al.*, 1968) and asymmetry parameters: Δ=25.4 (4), φ_m_=42.7 (2)°, ΔC_s_(14)=4.8 (3) and ΔC_2_(13,14)=14.9 (3)°]. The pseudo-torsion angle C19–C10···C13–C18 of 2.2 (2)° indicates that the molecule is only slightly twisted. The 6-methylene group is in a beta equatorial position with an angle of 63.8 (2)°. Due to the acidic character of hydrogen atoms close to C═C or C═O double bonds, cohesion of the crystal can be attributed to a net of two C–H···O pseudohydrogen bonds, namely C2–H2A···O17 and C66–H66A···O3, connecting molecules aligned almost along [101], respectively head to tail and head to head.

### Experimental

6-Methylenandrost-4-ene-3,17-dione was prepared according to a described procedure (Annen *et al.*, 1982) as follows. A suspension of anhydrous sodium acetate (1.0 g, 12.19 mmol) in dry chloroform (30.0 cm^3^) containing formaldehyde dimethyl acetal (30.0 cm^3^, 340.0 mmol) and phosphoryl choride (1.9 cm^3^, 20.0 mmol) was heated at reflux for 1 h. Androstenedione (773.5 mg, 2.70 mmol) was then added and the mixture was supplemented dropwise with phosphoryl choride (1.9 cm^3^, 20.0 mmol) over a period of 3 h 30 min. The reaction mixture was subsequently refluxed under nitrogen for 10 h, after which was allowed to cool to room temperature. A saturated aqueous solution of sodium carbonate was then added under vigorous stirring until the aqueous layer became alkaline. This mixture was extracted with chloroform (200 cm^3^) and then the organic phase was washed with water (4x100 cm^3^), dried over anhydrous MgSO_4_, filtered and concentrated to dryness. The resulting residue was purified by a silica gel 60 column chromatography (hexane/diethyl ether) affording the pure 6-methylenandrost-4-ene-3,17-dione (134.8 mg, 17%). Suitable crystals for X-ray studies were grown from slow evaporation from acetone/n-hexane: Mp. 435–437 K [lit 440 K (Annen *et al.*, 1982)]; IR ν_max_ (NaCl plates, CHCl_3_) cm^-1^: 3084 (=C–H), 1738 (C_17_=O), 1671 (C_3_=O), 1599 (C═C); ^1^H NMR (600 MHz, CDCl_3_): δ 0.78 (3*H*, *s*, 18–H_3_), 1.00 (3*H*, *s*, 19–H_3_), 4.87 (1*H*, *t*, ═CH_2_), 4.97 (1*H*, *t*, ═CH_2_), 5.79 (1*H*, *s*, 4–H); ^13^C NMR (150 MHz, CDCl_3_): δ 11.5 (C18), 14.9 (C19), 18.2, 19.5, 29.0, 31.6, 32.9, 33.0, 33.6, 36.6, 36.9, 45.3, 48.9, 50.3, 112.4 (═CH_2_), 119.6 (C4), 143.2 (C6), 166.3 (C5), 197.4 (C3), 217.8 (C17).

### Refinement

All hydrogen atoms were refined as riding on their parent atoms. Number of Friedel pairs measured: 1606 (45%). Due to the lack of any strong anomalous scatterer atom at the Mo Kα wavelength, refinement of Flack parameter was inconclusive. However the absolute configuration of the molecule is known from the synthetic route.

### Crystal data

**Table:**

C_20_H_26_O_2_	*F*(000) = 324
*M**_r_* = 298.41	*D*_x_ = 1.170 Mg m^−^^3^
Monoclinic, *P*2_1_	Mo *K*α radiation, λ = 0.71073 Å
*a* = 9.2343 (4) Å	Cell parameters from 4974 reflections
*b* = 8.7162 (4) Å	θ = 3.0–22.5°
*c* = 11.0798 (5) Å	µ = 0.07 mm^−^^1^
β = 108.197 (2)°	*T* = 293 K
*V* = 847.19 (7) Å^3^	Prism, colourless
*Z* = 2	0.24 × 0.17 × 0.05 mm

### Data collection

**Table:**

Bruker APEX CCD diffractometer	1979 independent reflections
Radiation source: fine-focus sealed tube	1433 reflections with *I* > 2σ(*I*)
Graphite monochromator	*R*_int_ = 0.036
φ and ω scans	θ_max_ = 28.2°, θ_min_ = 3.4°
Absorption correction: multi-scan (*SADABS*; Sheldrick, 2000)	*h* = −11→11
*T*_min_ = 0.835, *T*_max_ = 0.996	*k* = −11→11
18560 measured reflections	*l* = −14→14

### Refinement

**Table:**

Refinement on *F*^2^	Primary atom site location: structure-invariant direct methods
Least-squares matrix: full	Secondary atom site location: difference Fourier map
*R*[*F*^2^ > 2σ(*F*^2^)] = 0.040	Hydrogen site location: inferred from neighbouring sites
*wR*(*F*^2^) = 0.100	H-atom parameters constrained
*S* = 1.02	*w* = 1/[σ^2^(*F*_o_^2^) + (0.0554*P*)^2^ + 0.0289*P*] where *P* = (*F*_o_^2^ + 2*F*_c_^2^)/3
1979 reflections	(Δ/σ)_max_ < 0.001
201 parameters	Δρ_max_ = 0.14 e Å^−^^3^
1 restraint	Δρ_min_ = −0.13 e Å^−^^3^

### Special details

**Table:**

Geometry. All e.s.d.'s (except the e.s.d. in the dihedral angle between two l.s. planes) are estimated using the full covariance matrix. The cell e.s.d.'s are taken into account individually in the estimation of e.s.d.'s in distances, angles and torsion angles; correlations between e.s.d.'s in cell parameters are only used when they are defined by crystal symmetry. An approximate (isotropic) treatment of cell e.s.d.'s is used for estimating e.s.d.'s involving l.s. planes.
Refinement. Refinement of *F*^2^ against ALL reflections. The weighted *R*-factor *wR* and goodness of fit *S* are based on *F*^2^, conventional *R*-factors *R* are based on *F*, with *F* set to zero for negative *F*^2^. The threshold expression of *F*^2^ > σ(*F*^2^) is used only for calculating *R*-factors(gt) *etc*. and is not relevant to the choice of reflections for refinement. *R*-factors based on *F*^2^ are statistically about twice as large as those based on *F*, and *R*- factors based on ALL data will be even larger.

### Fractional atomic coordinates and isotropic or equivalent isotropic displacement parameters (Å^2^)

**Table:**

	*x*	*y*	*z*	*U*_iso_*/*U*_eq_	
O3	0.4355 (3)	0.2091 (3)	−0.50154 (16)	0.0901 (7)	
O17	0.8776 (2)	0.1624 (3)	0.50156 (17)	0.0859 (7)	
C1	0.6765 (3)	0.0835 (3)	−0.1938 (2)	0.0482 (6)	
H1A	0.7557	0.0070	−0.1627	0.058*	
H1B	0.5887	0.0510	−0.1700	0.058*	
C2	0.6322 (3)	0.0896 (3)	−0.3384 (2)	0.0587 (7)	
H2A	0.7234	0.1037	−0.3628	0.070*	
H2B	0.5867	−0.0076	−0.3730	0.070*	
C3	0.5234 (3)	0.2148 (3)	−0.3938 (2)	0.0579 (7)	
C4	0.5301 (3)	0.3509 (3)	−0.31480 (19)	0.0519 (6)	
H4	0.4632	0.4312	−0.3481	0.062*	
C5	0.6281 (3)	0.3660 (3)	−0.19633 (19)	0.0406 (5)	
C6	0.6402 (3)	0.5144 (3)	−0.12840 (19)	0.0429 (5)	
C7	0.6506 (3)	0.5032 (3)	0.00948 (19)	0.0466 (6)	
H7A	0.5514	0.4757	0.0158	0.056*	
H7B	0.6779	0.6028	0.0491	0.056*	
C8	0.7671 (3)	0.3855 (3)	0.08090 (18)	0.0393 (5)	
H8	0.8690	0.4198	0.0832	0.047*	
C9	0.7337 (3)	0.2294 (3)	0.01266 (18)	0.0366 (5)	
H9	0.6293	0.2027	0.0087	0.044*	
C10	0.7340 (2)	0.2374 (2)	−0.12870 (18)	0.0393 (5)	
C11	0.8350 (3)	0.1008 (3)	0.0884 (2)	0.0539 (6)	
H11A	0.9384	0.1168	0.0871	0.065*	
H11B	0.7999	0.0036	0.0468	0.065*	
C12	0.8365 (3)	0.0905 (3)	0.2274 (2)	0.0568 (7)	
H12A	0.7370	0.0584	0.2302	0.068*	
H12B	0.9107	0.0146	0.2722	0.068*	
C13	0.8765 (3)	0.2447 (3)	0.29190 (19)	0.0473 (6)	
C14	0.7637 (3)	0.3646 (3)	0.21692 (19)	0.0423 (5)	
H14	0.6623	0.3240	0.2093	0.051*	
C15	0.7863 (3)	0.5024 (4)	0.3071 (2)	0.0586 (7)	
H15A	0.6977	0.5690	0.2840	0.070*	
H15B	0.8756	0.5616	0.3079	0.070*	
C16	0.8078 (4)	0.4237 (4)	0.4357 (2)	0.0741 (9)	
H16A	0.8849	0.4765	0.5028	0.089*	
H16B	0.7130	0.4240	0.4561	0.089*	
C17	0.8573 (3)	0.2610 (4)	0.4218 (2)	0.0597 (7)	
C18	1.0444 (3)	0.2877 (4)	0.3115 (2)	0.0665 (8)	
H18A	1.1098	0.2100	0.3615	0.100*	
H18B	1.0667	0.3845	0.3546	0.100*	
H18C	1.0611	0.2953	0.2304	0.100*	
C19	0.8931 (3)	0.2731 (3)	−0.1379 (2)	0.0536 (6)	
H19A	0.8879	0.2794	−0.2257	0.080*	
H19B	0.9625	0.1930	−0.0971	0.080*	
H19C	0.9282	0.3691	−0.0968	0.080*	
C66	0.6447 (3)	0.6472 (3)	−0.1846 (2)	0.0609 (7)	
H66A	0.6403	0.6498	−0.2696	0.073*	
H66B	0.6523	0.7382	−0.1391	0.073*	

### Atomic displacement parameters (Å^2^)

**Table:**

	*U*^11^	*U*^22^	*U*^33^	*U*^12^	*U*^13^	*U*^23^
O3	0.1235 (18)	0.0890 (16)	0.0410 (9)	−0.0040 (15)	0.0016 (11)	−0.0116 (10)
O17	0.0974 (16)	0.1097 (18)	0.0578 (10)	0.0235 (14)	0.0349 (10)	0.0346 (12)
C1	0.0620 (15)	0.0377 (14)	0.0482 (12)	−0.0032 (12)	0.0218 (11)	−0.0054 (11)
C2	0.0806 (18)	0.0504 (16)	0.0498 (13)	−0.0085 (15)	0.0272 (12)	−0.0157 (12)
C3	0.0765 (18)	0.0587 (17)	0.0386 (11)	−0.0084 (15)	0.0182 (12)	−0.0052 (12)
C4	0.0636 (15)	0.0513 (15)	0.0383 (11)	0.0048 (14)	0.0121 (10)	0.0033 (12)
C5	0.0513 (14)	0.0367 (13)	0.0371 (10)	−0.0006 (11)	0.0188 (10)	0.0038 (10)
C6	0.0525 (14)	0.0361 (13)	0.0389 (11)	0.0049 (12)	0.0125 (10)	0.0032 (10)
C7	0.0623 (15)	0.0358 (13)	0.0402 (11)	0.0095 (13)	0.0138 (10)	−0.0020 (10)
C8	0.0412 (12)	0.0393 (14)	0.0362 (9)	0.0002 (10)	0.0103 (9)	0.0002 (9)
C9	0.0408 (12)	0.0322 (13)	0.0376 (10)	0.0003 (10)	0.0133 (9)	0.0017 (9)
C10	0.0488 (14)	0.0333 (13)	0.0385 (10)	−0.0033 (11)	0.0174 (9)	−0.0018 (9)
C11	0.0662 (16)	0.0441 (16)	0.0491 (12)	0.0100 (13)	0.0146 (11)	0.0050 (11)
C12	0.0657 (17)	0.0513 (17)	0.0520 (13)	0.0094 (14)	0.0161 (11)	0.0140 (12)
C13	0.0435 (13)	0.0575 (17)	0.0395 (11)	0.0015 (12)	0.0111 (10)	0.0080 (11)
C14	0.0407 (12)	0.0480 (14)	0.0367 (10)	0.0015 (12)	0.0098 (9)	−0.0005 (11)
C15	0.0689 (17)	0.0625 (17)	0.0412 (12)	0.0014 (15)	0.0124 (11)	−0.0081 (12)
C16	0.085 (2)	0.095 (2)	0.0402 (13)	0.0036 (19)	0.0172 (13)	−0.0058 (15)
C17	0.0503 (15)	0.086 (2)	0.0418 (12)	0.0018 (15)	0.0129 (10)	0.0095 (14)
C18	0.0447 (15)	0.093 (2)	0.0583 (15)	0.0041 (15)	0.0103 (11)	0.0095 (15)
C19	0.0528 (14)	0.0550 (16)	0.0605 (14)	−0.0022 (13)	0.0284 (11)	−0.0029 (12)
C66	0.086 (2)	0.0426 (15)	0.0537 (14)	0.0026 (15)	0.0218 (13)	0.0052 (12)

### Geometric parameters (Å, º)

**Table:**

O3—C3	1.217 (3)	C11—C12	1.539 (3)
O17—C17	1.205 (3)	C11—H11A	0.9700
C1—C2	1.526 (3)	C11—H11B	0.9700
C1—C10	1.537 (3)	C12—C13	1.512 (4)
C1—H1A	0.9700	C12—H12A	0.9700
C1—H1B	0.9700	C12—H12B	0.9700
C2—C3	1.481 (4)	C13—C17	1.511 (3)
C2—H2A	0.9700	C13—C14	1.525 (3)
C2—H2B	0.9700	C13—C18	1.543 (4)
C3—C4	1.464 (4)	C14—C15	1.534 (4)
C4—C5	1.348 (3)	C14—H14	0.9800
C4—H4	0.9300	C15—C16	1.538 (4)
C5—C6	1.483 (3)	C15—H15A	0.9700
C5—C10	1.521 (3)	C15—H15B	0.9700
C6—C66	1.322 (4)	C16—C17	1.512 (5)
C6—C7	1.504 (3)	C16—H16A	0.9700
C7—C8	1.518 (3)	C16—H16B	0.9700
C7—H7A	0.9700	C18—H18A	0.9600
C7—H7B	0.9700	C18—H18B	0.9600
C8—C14	1.528 (3)	C18—H18C	0.9600
C8—C9	1.540 (3)	C19—H19A	0.9600
C8—H8	0.9800	C19—H19B	0.9600
C9—C11	1.531 (3)	C19—H19C	0.9600
C9—C10	1.568 (3)	C66—H66A	0.9300
C9—H9	0.9800	C66—H66B	0.9300
C10—C19	1.536 (3)		
			
C2—C1—C10	113.72 (19)	C9—C11—H11B	108.8
C2—C1—H1A	108.8	C12—C11—H11B	108.8
C10—C1—H1A	108.8	H11A—C11—H11B	107.7
C2—C1—H1B	108.8	C13—C12—C11	110.2 (2)
C10—C1—H1B	108.8	C13—C12—H12A	109.6
H1A—C1—H1B	107.7	C11—C12—H12A	109.6
C3—C2—C1	112.7 (2)	C13—C12—H12B	109.6
C3—C2—H2A	109.0	C11—C12—H12B	109.6
C1—C2—H2A	109.0	H12A—C12—H12B	108.1
C3—C2—H2B	109.0	C17—C13—C12	116.7 (2)
C1—C2—H2B	109.0	C17—C13—C14	101.05 (19)
H2A—C2—H2B	107.8	C12—C13—C14	109.11 (18)
O3—C3—C4	120.7 (3)	C17—C13—C18	104.76 (19)
O3—C3—C2	122.3 (2)	C12—C13—C18	111.6 (2)
C4—C3—C2	116.9 (2)	C14—C13—C18	113.3 (2)
C5—C4—C3	123.5 (2)	C13—C14—C8	113.47 (18)
C5—C4—H4	118.3	C13—C14—C15	104.54 (17)
C3—C4—H4	118.3	C8—C14—C15	120.6 (2)
C4—C5—C6	120.0 (2)	C13—C14—H14	105.7
C4—C5—C10	122.7 (2)	C8—C14—H14	105.7
C6—C5—C10	117.22 (17)	C15—C14—H14	105.7
C66—C6—C5	122.25 (18)	C14—C15—C16	101.9 (2)
C66—C6—C7	122.3 (2)	C14—C15—H15A	111.4
C5—C6—C7	115.46 (19)	C16—C15—H15A	111.4
C6—C7—C8	112.53 (18)	C14—C15—H15B	111.4
C6—C7—H7A	109.1	C16—C15—H15B	111.4
C8—C7—H7A	109.1	H15A—C15—H15B	109.3
C6—C7—H7B	109.1	C17—C16—C15	106.3 (2)
C8—C7—H7B	109.1	C17—C16—H16A	110.5
H7A—C7—H7B	107.8	C15—C16—H16A	110.5
C7—C8—C14	111.40 (17)	C17—C16—H16B	110.5
C7—C8—C9	109.92 (16)	C15—C16—H16B	110.5
C14—C8—C9	108.45 (18)	H16A—C16—H16B	108.7
C7—C8—H8	109.0	O17—C17—C13	126.4 (3)
C14—C8—H8	109.0	O17—C17—C16	125.3 (2)
C9—C8—H8	109.0	C13—C17—C16	108.3 (2)
C11—C9—C8	112.54 (16)	C13—C18—H18A	109.5
C11—C9—C10	112.90 (17)	C13—C18—H18B	109.5
C8—C9—C10	112.83 (16)	H18A—C18—H18B	109.5
C11—C9—H9	105.9	C13—C18—H18C	109.5
C8—C9—H9	105.9	H18A—C18—H18C	109.5
C10—C9—H9	105.9	H18B—C18—H18C	109.5
C5—C10—C19	107.41 (18)	C10—C19—H19A	109.5
C5—C10—C1	109.70 (17)	C10—C19—H19B	109.5
C19—C10—C1	110.02 (19)	H19A—C19—H19B	109.5
C5—C10—C9	108.90 (16)	C10—C19—H19C	109.5
C19—C10—C9	112.03 (17)	H19A—C19—H19C	109.5
C1—C10—C9	108.75 (16)	H19B—C19—H19C	109.5
C9—C11—C12	113.8 (2)	C6—C66—H66A	120.0
C9—C11—H11A	108.8	C6—C66—H66B	120.0
C12—C11—H11A	108.8	H66A—C66—H66B	120.0
			
C10—C1—C2—C3	−52.5 (3)	C8—C9—C10—C19	66.5 (2)
C1—C2—C3—O3	−153.9 (3)	C11—C9—C10—C1	59.3 (2)
C1—C2—C3—C4	28.6 (3)	C8—C9—C10—C1	−171.65 (17)
O3—C3—C4—C5	−177.9 (3)	C8—C9—C11—C12	50.9 (3)
C2—C3—C4—C5	−0.4 (4)	C10—C9—C11—C12	−180.0 (2)
C3—C4—C5—C6	172.8 (2)	C9—C11—C12—C13	−53.2 (3)
C3—C4—C5—C10	−4.8 (4)	C11—C12—C13—C17	170.15 (19)
C4—C5—C6—C66	−43.6 (3)	C11—C12—C13—C14	56.5 (3)
C10—C5—C6—C66	134.1 (2)	C11—C12—C13—C18	−69.4 (2)
C4—C5—C6—C7	137.7 (2)	C17—C13—C14—C8	174.9 (2)
C10—C5—C6—C7	−44.6 (3)	C12—C13—C14—C8	−61.5 (2)
C66—C6—C7—C8	−130.9 (3)	C18—C13—C14—C8	63.4 (3)
C5—C6—C7—C8	47.7 (3)	C17—C13—C14—C15	41.7 (2)
C6—C7—C8—C14	−174.15 (19)	C12—C13—C14—C15	165.22 (19)
C6—C7—C8—C9	−53.9 (3)	C18—C13—C14—C15	−69.8 (2)
C7—C8—C9—C11	−172.96 (18)	C7—C8—C14—C13	178.7 (2)
C14—C8—C9—C11	−51.0 (2)	C9—C8—C14—C13	57.6 (2)
C7—C8—C9—C10	57.8 (2)	C7—C8—C14—C15	−56.3 (3)
C14—C8—C9—C10	179.86 (17)	C9—C8—C14—C15	−177.35 (19)
C4—C5—C10—C19	101.4 (2)	C13—C14—C15—C16	−39.5 (2)
C6—C5—C10—C19	−76.3 (2)	C8—C14—C15—C16	−168.6 (2)
C4—C5—C10—C1	−18.2 (3)	C14—C15—C16—C17	21.5 (3)
C6—C5—C10—C1	164.16 (18)	C12—C13—C17—O17	34.0 (4)
C4—C5—C10—C9	−137.1 (2)	C14—C13—C17—O17	152.1 (3)
C6—C5—C10—C9	45.3 (2)	C18—C13—C17—O17	−90.0 (3)
C2—C1—C10—C5	46.0 (3)	C12—C13—C17—C16	−146.0 (2)
C2—C1—C10—C19	−72.0 (2)	C14—C13—C17—C16	−27.8 (3)
C2—C1—C10—C9	164.96 (19)	C18—C13—C17—C16	90.0 (3)
C11—C9—C10—C5	178.85 (19)	C15—C16—C17—O17	−176.0 (3)
C8—C9—C10—C5	−52.1 (2)	C15—C16—C17—C13	3.9 (3)
C11—C9—C10—C19	−62.5 (2)	C19—C10—C13—C18	2.2 (2)

### Hydrogen-bond geometry (Å, º)

**Table:**

*D*—H···*A*	*D*—H	H···*A*	*D*···*A*	*D*—H···*A*
C2—H2*A*···O17^i^	0.97	2.43	3.345 (3)	158
C66—H66*A*···O3^ii^	0.93	2.47	3.365 (3)	163

Symmetry codes: (i) *x*, *y*, *z*−1; (ii) −*x*+1, *y*+1/2, −*z*−1.
